# Using Expert Elicitation to Abridge the Welfare Quality® Protocol for Monitoring the Most Adverse Dairy Cattle Welfare Impairments

**DOI:** 10.3389/fvets.2021.634470

**Published:** 2021-05-28

**Authors:** Frank A. M. Tuyttens, Sophie de Graaf, Sine Norlander Andreasen, Alice de Boyer des Roches, Frank J. C. M. van Eerdenburg, Marie J. Haskell, Marlene K. Kirchner, Luc. Mounier, Miroslav Kjosevski, Jo Bijttebier, Ludwig Lauwers, Wim Verbeke, Bart Ampe

**Affiliations:** ^1^Animal Sciences Unit, Institute for Agricultural and Fisheries Research (ILVO), Merelbeke, Belgium; ^2^Department of Nutrition, Genetics and Ethology, Faculty of Veterinary Medicine, Ghent University, Merelbeke, Belgium; ^3^Department of Agricultural Economics, Ghent University, Ghent, Belgium; ^4^Department of Veterinary and Animal Sciences, University of Copenhagen, Frederiksberg, Denmark; ^5^Université Clermont Auvergne, INRAE, VetAgro Sup, UMR Herbivores, Saint-Genès-Champanelle, France; ^6^Department of Veterinary and Animal Sciences, Section of Animal Welfare and Disease Control, University of Copenhagen, Frederiksberg, Denmark; ^7^Scotland's Rural College, Department of Population Health Sciences, Section Farm Animal Health, Faculty of Veterinary Medicine, Utrecht University, Utrecht, Netherlands; ^8^Animal Behavior and Welfare, Animal and Veterinary Sciences, SRUC, Edinburgh, United Kingdom; ^9^Animal Welfare Center, Faculty of Veterinary Medicine, Ss. Cyril and Methodius University in Skopje, Skopje, North Macedonia

**Keywords:** animal welfare, dairy cattle, integration, welfare assessment, compensation, aggregation, index

## Abstract

The Welfare Quality® consortium has developed and proposed standard protocols for monitoring farm animal welfare. The uptake of the dairy cattle protocol has been below expectation, however, and it has been criticized for the variable quality of the welfare measures and for a limited number of measures having a disproportionally large effect on the integrated welfare categorization. Aiming for a wide uptake by the milk industry, we revised and simplified the Welfare Quality® protocol into a user-friendly tool for cost- and time-efficient on-farm monitoring of dairy cattle welfare with a minimal number of key animal-based measures that are aggregated into a continuous (and thus discriminative) welfare index (WI). The inevitable subjective decisions were based upon expert opinion, as considerable expertise about cattle welfare issues and about the interpretation, importance, and validity of the welfare measures was deemed essential. The WI is calculated as the sum of the severity score (i.e., how severely a welfare problem affects cow welfare) multiplied with the herd prevalence for each measure. The selection of measures (lameness, leanness, mortality, hairless patches, lesions/swellings, somatic cell count) and their severity scores were based on expert surveys (14–17 trained users of the Welfare Quality® cattle protocol). The prevalence of these welfare measures was assessed in 491 European herds. Experts allocated a welfare score (from 0 to 100) to 12 focus herds for which the prevalence of each welfare measure was benchmarked against all 491 herds. Quadratic models indicated a high correspondence between these subjective scores and the WI (*R*^2^ = 0.91). The WI allows both numerical (0–100) as a qualitative (“not classified” to “excellent”) evaluation of welfare. Although it is sensitive to those welfare issues that most adversely affect cattle welfare (as identified by EFSA), the WI should be accompanied with a disclaimer that lists adverse or favorable effects that cannot be detected adequately by the current selection of measures.

## Introduction

A tool to correctly assess and monitor animal welfare is key to many initiatives to improve the welfare of livestock (1). Obviously, the characteristics of this monitoring tool depend on how it is to be applied. For example, the tool may be very elaborate, refined, high tech, and comprehensive if it is to be used in experimental animal welfare research or for in-depth assessments of a limited number of focal herds by a multidisciplinary team of highly trained specialists. The focus of the current study, however, is on a tool that is to be taken up widely by the food industry at large (e.g., for an animal welfare label on food products). For this type of application, the logistic feasibility, the costs, and the user-friendliness are major constraints. At the same time, as socioeconomic stakes can be high, decisions about the animal welfare status allocated to herds or food products ought to be transparent, non-disputable, and accepted as valid by the main stakeholders (e.g., farmer, auditor, retailer, consumer).

Balancing these logistic and scientific requirements is a huge challenge. As a multidimensional societal concept, the number of ways that the welfare of livestock can be affected positively or negatively, and how these effects can be assessed, is very diverse and almost endless. The scientific ambition to accurately document any small change in the status of any of these multiple animal welfare aspects is poorly compatible with the industry demand that the tool is cost efficient and easy to implement. Hence, choices will need to be made about which aspects of welfare to include and about the resolution by which these will be documented. These choices will be subjective to some degree because the conception of animal welfare is partly values based, and people differ in what they consider important or desirable for animals to have a good life (2).

Another characteristic of the monitoring tool that depends on the intended application concerns the need to aggregate the information from the individual welfare measures into an integrated, balanced overall welfare index (WI). Such aggregation may be redundant in case the tool is used to provide farm-specific feedback on how certain welfare problems in a herd could be addressed. However, it is essential for the purpose of the tool developed in this study, namely, to inform consumers about the general welfare status of the animals from which food is derived (1). In fact, aggregating data from various welfare measures into a WI reflecting the overall welfare status of the herd is one of the most difficult challenges in animal welfare science (3). As there is no “gold standard” for overall herd welfare, aggregating data on various welfare measures into an overall index again requires some degree of subjectivity (4).

Standardized methodologies for assessing the welfare of various categories of farm animals, including broiler chickens, laying hens, growing pigs, sows, veal calves, and dairy cattle, were developed in the European Welfare Quality® (WQ) project (5). The WQ protocols have been praised for being very comprehensive and for the implementation of a hierarchical approach to integrate data on a multitude of predominantly animal-based welfare measures enabling the assignment of farms or herds to one of the four overall welfare categories (not classified, acceptable, enhanced, and excellent). Although issues about consistency over time (6–9) and about reliance on complete and standardized farm/slaughterhouse records (10–12) have been raised, the WQ protocols have been criticized mainly with regard to the (i) the feasibility [mainly labor costs per farm, e.g., (11, 13)], (ii) the variable quality of the welfare measures included in the protocol (8, 10, 14), and (iii) the way these measures are aggregated into an overall WI (15–21). Indeed, uptake of the WQ protocols by the authorities and food industry at large for improving and better marketing of farm animal welfare has been below expectation. Although stakeholders have expressed interest in welfare monitoring of various types of farm animals, they have emphasized that the labor demand of about one farm or herd per day per certified assessor needs to be reduced. de Jong et al. (11) have addressed these industry concerns by proposing time-saving simplifications to the WQ broiler chicken protocol but—to our knowledge—no such modifications have been shown promising for the other protocols. This is particularly needed for the dairy cattle protocol as it takes up to 4.4–7.7 h to complete for a herd of 25–200 cows, respectively, excluding the time needed for making the appointment and for travel (22).

Criticisms on welfare measures often relate to their poor reliability, validity, or feasibility (10, 11, 13, 14). There is a growing consensus now that animal-based measures are preferred for directly assessing the outcome of the complex effects of the environment and management on the animal's actual state of welfare (1, 23, 24). Although one of the novel characteristics of the WQ protocols was the emphasis on animal-based measures, the WQ protocols also include resource- or management-based measures that have been criticized for describing the potential or risk for good or bad welfare rather than directly measuring the welfare status itself. The dairy cattle protocol, for example, relies on resource-based measures for assessing 3 of the 12 welfare criteria (water availability and cleanliness for the criterion absence of prolonged thirst, tethering for the criterion ease of movement, and pasture access for the criterion expression of other behaviors). It is particularly worrying that sensitivity analyses have revealed that a limited number of (often resource-based) measures seem to have a disproportionally large effect on the overall welfare categorization [e.g., 88% of the overall dairy cattle welfare categorization is predicted by water availability and cleanliness (17)], whereas some key (often animal-based) measures such as lameness and mortality have a negligible effect (16–18, 21). This appears to be an unwanted side effect of the very complex and hard-to-understand (and hence poorly transparent to most end-users) integration method, which was needed to aggregate so many measures of different scales with different thresholds.

Aiming for a wide uptake by the milk industry, in the current study, we revised and simplified the WQ dairy cattle protocol with a view to (i) drastically reduce the time needed to complete an assessment, (ii) make use of a minimal number of key animal-based measures, and (iii) transparently aggregate these measures into a continuous (and thus discriminative) WI. We describe and illustrate the steps in the development of this revised and simplified protocol for quantifying the level of herd welfare, albeit without claiming to be exhaustive. The WI is based upon the intuitively sensible method of Burow et al. (25) in which the relative weight of each welfare measure depends on its severity score (expert judgement of how severely a given welfare problem affects the welfare of an individual cow) multiplied by the herd prevalence for that measure. Moreover, we investigate the extent to which the integration method should allow compensation of poor scores with better scores. In some studies (4), it is argued that such compensation should be restrained, as good results on one aspect cannot compensate for poor scores on other aspects (e.g., having a good body condition score cannot compensate for being severely lame). Other studies, however, indicate that compensation between welfare aspects may be possible [reviewed by Leknes and Tracey (26)]. At present, there is little evidence that compensation reduction is warranted, let alone what type of compensation-reduction method best corresponds with expert opinion. The latter is examined in one of the proposed steps in this study. Some of the steps inevitably demand subjective decisions. These were based upon expert (defined as an animal scientist trained to use the WQ dairy cattle protocol) opinion, as considerable expertise about cattle welfare issues and about the interpretation, importance, and validity of the welfare measures was deemed essential. For this study we opted not to involve people without in-depth knowledge and expertise in dairy cattle welfare and the measures involved because of doubts about their ability to adequately balance the importance of different welfare measures. Indeed, the relative importance that ought to be allocated to a given welfare measure could depend on how exactly it is measured on-farm (e.g., selection of and size of the sample, to what extent confounding factors may influence the measures, objectivity of the measure). Moreover, it has been shown that detailed information on how data on welfare measures is collected on-farm can significantly influence the relative weights they are given by experts (27). Even for dairy cattle welfare experts, it can be a daunting task to make decisions about overall welfare status by integrating the scores of the various measures in such a way that the outcome reflects the range of what can be expected among real farms and allows realistic differentiation between these farms. Expert welfare scoring of herds was, therefore, based on a large database of WQ data that reflect a wide range of dairy herd types in Europe and thereby ensuring a substantial but realistic spread in observed values.

## Materials and Methods

Our approach to revise and simplify the WQ dairy cattle protocol involved five steps. The same steps can be used to revise and simplify the other WQ protocols or to add additional welfare measures if this would be deemed desirable. The first four steps inevitably require subjective decisions for which experts with knowledge of the WQ dairy cattle protocol were consulted. We emailed 31 researchers who were known to the authors, to our network, or to the Welfare Quality Network to have been trained to use the WQ dairy cattle protocol. These trained users were in turn asked to provide contact details of any additional animal welfare scientists who would be suitable (i.e., trained to use the WQ protocol). Fourteen declined the invitation to participate because they could not fill out the survey in time or did not respond. All experts who agreed to participate in the current study had experience with the WQ protocol for dairy cattle (i.e., were trained to perform the WQ protocol for dairy cattle and had used it to assess the welfare of dairy herds), were animal scientists, and had authored at least one peer-reviewed scientific paper about dairy cattle welfare involving the WQ protocol. Although we did not select for this, all participating experts were from Europe (the WQ protocols are used predominantly in Europe), and a total of eight nationalities were represented (British, Spanish, Macedonian, Dutch, Finnish, Austrian, German, and French). No experts whose input was used in the analyses were involved in creating the surveys.

Step 1 entails selecting animal-based welfare measures to be included in the protocol. At the core of Steps 2 and 3 is the WI. Based upon Burow et al. (25), the WI was constructed from perceived severity of welfare problems (“severity score”) and observed prevalence of these welfare problems. The severity scores for the various welfare measures were determined in Step 2 by asking the experts to score how severely each of the selected welfare problems (that are quantified by the selected measures) impairs the welfare of an animal. The following formula forms the basis to integrate data on selected welfare measures into one score:

$$\begin{array}{l} Welfare\;index\;score=\;\frac{1}{nm}\;\times \;\overset{nm}{\underset{m=1}{\sum }}Sm\times rPm \end{array}$$

Here, n represents “number,” m refers to “measure,” S represents the “severity score,” which ranged from 0 to 100, and rP refers to “relative prevalence,” which is calculated as prevalence per herd/prevalence at 97.5th percentile of that measure among all herds in the EU database. In the proposed formula, rP rather than absolute prevalence was used so each herd covered the same possible spectrum for each measure. Prevalence of the 97.5th percentile was set as the maximum for each measure score, to prevent an extreme prevalence value of single measures from having a disproportionately large influence on the score. Therefore, herds with values equal to or higher than the 97.5th percentile were automatically given the maximum measure score. This allowed for a uniform method to determine thresholds for the different compensation-reduction methods (CRMs) that were tested. To achieve a score on a scale of 0 (very poor welfare)−100 (excellent welfare) and to test various CRMs, the formula was complemented as follows:

$$\begin{array}{l} Welfare\;index\;score=100-\frac{100}{Smax\;}\times \overset{nm}{\underset{m=1}{\sum }}Sm\times rPm\;\times \;\text{C}\text{m} \end{array}$$

Here, Cm is the “compensation-reduction factor” for measure m (value between 1 and Cmax), and Smax is the sum of the products of Sm and the maximal compensation-reduction factor ($Smax=\;\overset{nm}{\underset{m=1}{\sum }}Sm\times Cmax$). To gain input for this formula, we performed two independent online surveys among the dairy cattle welfare experts. In Step 3, the WI is calculated, and correspondence with expert opinion is analyzed. Similarity between experts' welfare scores for several fictitious herds and integrated WI using the aforementioned formula with various CRMs is analyzed. Step 4 consists of interpreting the WI (what score indicates poor/good welfare). Step 5 comprises of checking to what degree the selected welfare measures are associated with factors that have the most severe impact on dairy cattle welfare. The five steps are elaborated below.

### Step 1: Selecting Welfare Measures

Welfare measures were selected from the WQ protocol for dairy cattle (22). We used three criteria for selecting measures: (1) they ought to be animal-based, (2) it must be possible to express them as a percentage to allow using the proposed WI-formula, and (3) they must be considered as important for dairy cattle welfare by the experts. The importance of the measures was based upon an online survey where 17 experts ranked all WQ measures (*n* = 27) on importance for the overall welfare status of a herd of dairy cattle. Although the experts were presumed familiar with each of these measures, the precise methodology could be consulted in the WQ protocol for the assessment of dairy cow welfare (www.welfarequalitynetwork.net). It was mentioned to the experts that for ranking (*inter alia*) reliability, validity, perceived relevance, and prevalence may be considered. Subsequently, we compared compliance of these selected measures with the outcomes of published studies in which expert opinion had been used as well to rank cattle welfare measures on importance (25, 28–30). Hence, in theory, measures could have been added in the case that the literature search would have revealed important animal-based measures that had not passed our initial selection (but this was not the case in our study).

### Step 2: Determining Severity Scores

To determine the severity scores for the selected measures, 14 of the same aforementioned 17 experts completed a second survey. In this second survey, they were asked to score how severely the welfare of an individual cow is affected by each of the six selected welfare impairments on a scale of 0 (totally not severe)−100 (extremely severe). The experts were informed that they may take (their perception of) both the degree and duration of suffering into account. In the ensuing Step 3, median severity scores were used in calculating the WI.

### Step 3: Calculating WI and Testing Coherence With Expert Opinion

**Figure 1:**
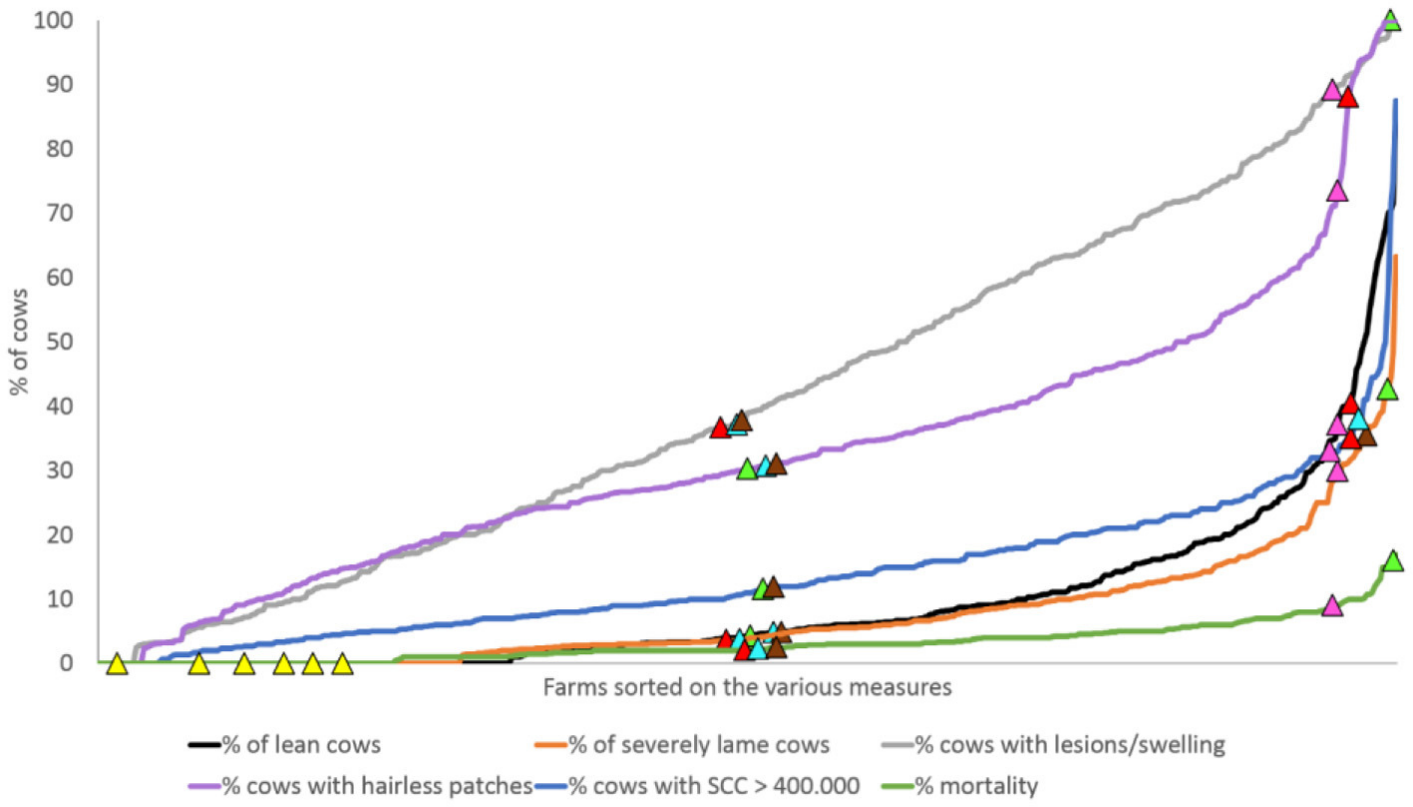
One of the graphs presented to experts in the second survey showing the distribution of all herds in the database (*n* = 491) for the six selected measures. Colored triangles mark six (of the 12) focus herds.

**Figure 2:**
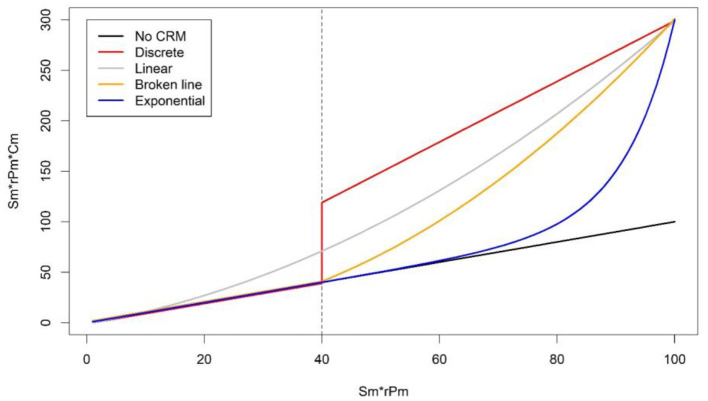
Illustration of the compensation reduction methods (except Veto) tested in this study with a maximal compensation of 3 and a threshold of 40. No compensation reduction method (CRM, black line) results in the diagonal (value before and after compensation is the same). Discrete gives no compensation reduction for measures up to a certain threshold of Sm*rPm, above which the Sm*rPm score is multiplied with maximum fixed value Cm. For linear CRM, Cm increases linearly with an increasing Sm*rPm score of the welfare measures. The broken line CRM gives no compensation reduction for measures up to a certain threshold of Sm*rPm, above which Cm increases in a linear manner. Exponential CRM increases Cm exponentially with an increasing Sm*rPm score of the welfare measures.

For checking correspondence between expert scores and aggregated WIs, in the subsequent part of the second survey, the 14 selected experts were presented with a graph showing the observed prevalence distribution of all selected welfare measures for 491 European herds that had been assessed using the WQ protocol (Figure 1). To reflect the current range present in Europe across various herding systems, existing WQ datasets were collated from seven European research institutes and included data from 10 countries [Macedonia, The Netherlands, France, Belgium, Scotland, Denmark, Romania, Northern Ireland, Spain, and Austria, more details in de Graaf et al. (20)]. In the graph, six “focus herds” were highlighted per expert (example: Figure 1; data shown in Table 1). These focus herds were fictitious but were based upon real herd data from the European dataset. In total, 12 focus herds were created to fit the following descriptions: (1) two herds that scored high in prevalence, taking the European dataset as a reference (indicating poor welfare) on all measures; (2) two herds that scored low (indicating good welfare) on all measures; (3) two herds that scored medium on one-half of the measures and high on the other half; (4) two herds that scored the other half of the measures medium and the other half high; (5) two herds that scored medium on all measures except for one (high for somatic cell count > 400,000), and (6) two herds that scored medium on all measures but high for one (high for severe lameness). High scoring measures in the latter two mentioned herds were chosen randomly from the selected measures. Highest prevalence belonged to the top 5% for all welfare measures, medium between 40 and 60%, and lowest scores were from the lowest 5%. Each expert was presented with six focus herds, one of the two for each category (Table 1). Experts were asked to allocate a welfare score to each focus herd they were presented with using a tagged visual analog scale from 0 to 100. Tags were “Not Classified (<20),” “Acceptable (20–55),” “Enhanced (55–80),” and “Excellent (>80),” following WQ categorization (22). Each of the 12 focus herds was thus scored by six to eight experts. Subsequently, the degree of correspondence between expert scores and WI's were calculated with varying CRMs. One of the tested CRMs was “veto,” where thresholds are defined for each measure above which a value cannot be compensated for. This is achieved by automatically attributing the worst possible welfare score to a herd, independent of the prevalence of other welfare problems. The other tested CRMs use various formulas to allocate increasingly more weight to worse scores on a certain measure. Tested formula in the current study were “Discrete,” “Linear,” “Broken line,” and “Exponential” and are illustrated in Figure 2. In addition, scores were calculated without CRM (“no CRM”), thus allowing full compensation between measures as default.

**Table 1:** Prevalences for the 6 selected dairy cattle welfare measures, for each of the 12 fictitious herds the experts (*n* = 14) allocated an integrated index score.

**Herd**	**Measure scores**	**Very lean**	**Severely lame**	**Lesions and swellings**	**Hairless patches**	**SCC > 400,000**	**Mortality**
1	All low^a^	0	0	3	3	0	0
2	All low^a^	0	0	0	0	0	0
3	All high^a^	46	33	92	92	38	10
4	All high^a^	37	30	90	74	33	9
5	Medium/high^a^^,^^b^	50	4	37	94	41	2
6	Medium/high^a^^,^^b^	41	4	37	88	35	2
7	High/medium^a^^,^^c^	4	32	92	30	11	10
8	High/medium^a^^,^^c^	4	44	100	30	11	16
9	Medium, high SCC^a^	4	4	37	30	35	2
10	Medium, high SCC^a^	4	4	37	30	38	2
11	Medium, high lameness^a^	5	34	39	30	11	2
12	Medium, high lameness^a^	4	33	37	30	11	2

a *Highest scores belonged to the top 5% of herds in the European dataset (n = 491), medium between 40 and 60%, and lowest scores were from the lowest 5% of herds*.

b *“% of too lean cows,” “somatic cell count (SCC) > 400,000,” and “nHP” were high*.

c *“% of cows with lesions,” “% of cows with severe lameness,” and “% of mortality” were high*.

For discrete, broken line, and veto CRM, a threshold at which compensation reduction starts needed to be determined. For all CRMs apart from veto, it also had to be determined what the maximum level of compensation reduction (Cmax) was. We checked which threshold value of S^*^rP (ranging between 5 and 70 in increments of 5) and which value for Cmax (set at between 1.5, 2, 3, 5, and 10) corresponded best with expert opinion based on model *R*^2^. For the 20 models with the highest *R*^2^, we calculated also the Akaike information criterion (AIC) and four additional metrics [root mean square error (RMSE), mean absolute difference, Liao's improved concordance correlation coefficient [ICCC, (31), and the Bland–Altman 95% limits of agreement [LOA, (32)] for quantifying the agreement between the model prediction and the experts' opinion. We ranked these 20 models according to the six agreement metrics and calculated the mean rank (giving equal weight to each of the six metrics). The model with the lowest mean rank was selected as the model (i.e., type of CRM) that provided the best fit with the opinion of the experts.

Statistical analyses were performed using the program R 3.2.2 (R Foundation for Statistical Computing, Vienna, Austria). Both linear and quadratic models were used to test correspondence between expert scoring and the integrated scores to determine if adding a CRM to the WI formula generated a better fit for varying thresholds and values of C. The Agreement Interval package was used to calculate the measures of agreement.

### Step 4: Interpreting the WI

To interpret the WI scores in terms of bad/medium/good welfare, we asked the experts to score overall welfare for the 12 focus herds on a tagged visual analog scale with labels for four welfare categories following WQ categorization (“not classified” from 0 to 20, “acceptable” from 20 to 55, “enhanced” from 55 to 80, and “excellent” from 80 to 100). To extrapolate thresholds of these welfare categories, we (scatter) plotted the expert scores against the WI scores for the 12 fictitious herds and added the best fitting curve. We then identified the three points where the best-fitting curve intersects with the WQ thresholds of the scale on which the experts scored (expert scores 20, 55, and 80).

### Step 5: Exhaustiveness Check

In Step 5, we assessed to what degree the selected measures are indicative of the “worst adverse effects” (factors that have the most severe impact) on dairy cattle welfare. For this end, we compared the selection of welfare measures with a list of worst adverse effects on dairy cattle welfare and associated animal-based welfare measures in a European Food Safety Authority (EFSA) report by Nielsen et al. (30). In this report, worst adverse effects were selected based upon several other EFSA reports (24, 33–37), Presi and Reist (38), Brenninkmeyer and Winckler (39), and expert opinion (Table 2).

**Table 2:** Summary of which of the “worst adverse effects” for dairy cattle welfare are associated with the selection of welfare measures in the current study based upon Nielsen et al. (30).

**Adverse effects**	**Associated welfare measures**
Foot disorders	Lameness, mortality, and lesions/swellings
Leg injuries	Lameness, lesions/swellings
Mortality (unassisted)	Mortality
Mortality (euthanasia)	Mortality
Exhaustion (prolonged metabolic demand)	Leanness, mortality, and lesions/swellings
Behavioral disruption—feeding (including social stress, pain, hunger, exhaustion, fear, and frustration)	Leanness, lameness
Behavioral disruption—rest (including too little rest, pain, and fear)	Lesions/swellings, lameness
Behavioral disruption—flooring/space (including fear, and pain)	Lesions/swellings, lameness
Thermal discomfort	No associations identified

## Results

### Step 1: Selecting Welfare Measures

**Figure 3:**
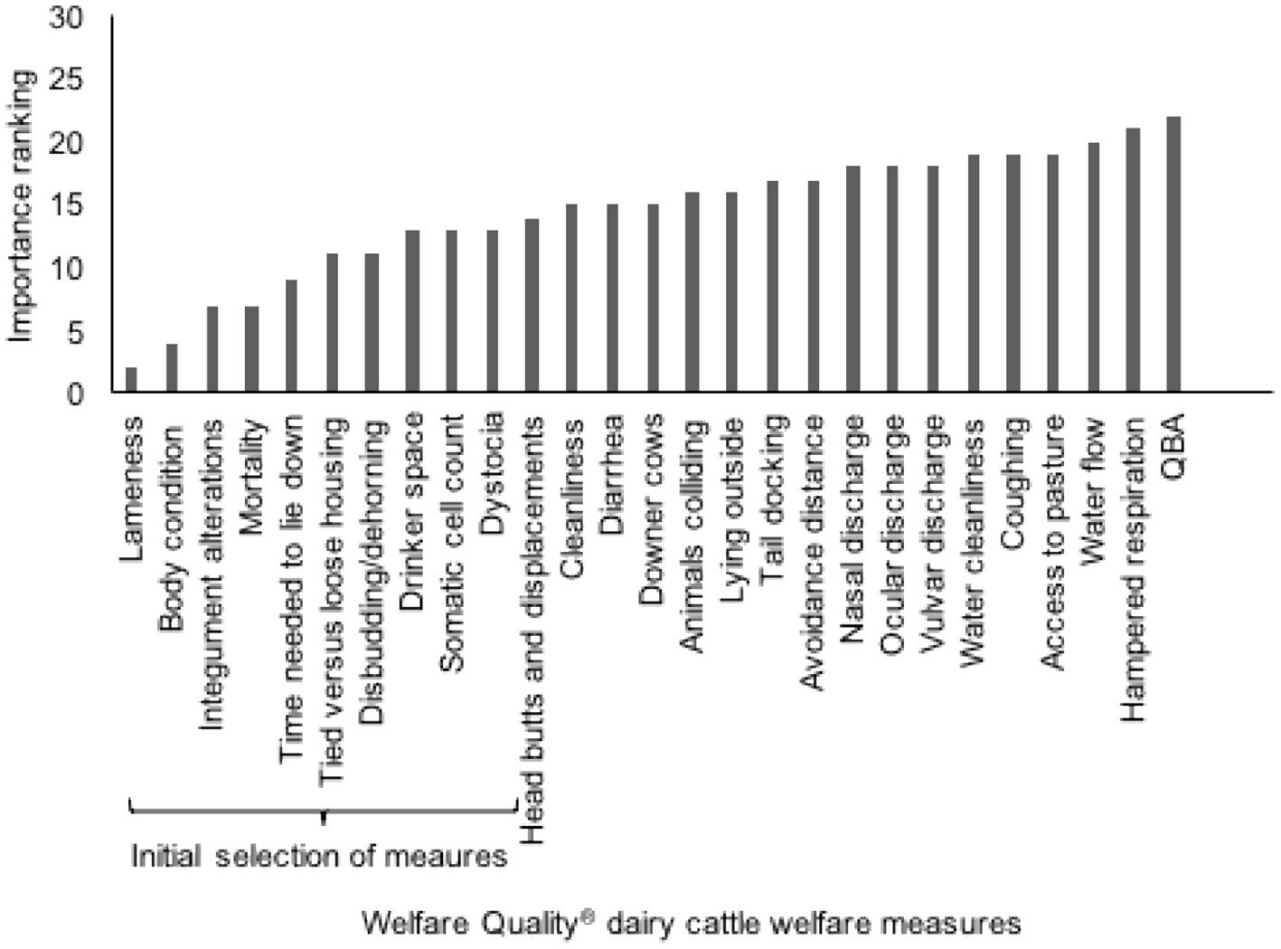
Median importance ranking for all WQ measures as judged by 16 dairy cattle welfare experts. Initial selection of 10 welfare measures for the WI indicated using accolade.

Highest median expert importance ranking for herd welfare was allocated to “lameness,” “leanness,” “mortality rate,” and “integument alterations,” which were therefore selected to be included in the protocol (Figure 3). The other measures among the top 10 ranked welfare measures were considered for inclusion as well: “time needed to lie down,” “tied vs. loose housing,” “disbudding/dehorning,” “drinker space,” “somatic cell count (SCC),” and “dystocia.” Only one of the latter measures (SCC >400,000 as an indicator of mastitis) met all selection criteria. Lameness is measured in WQ using a gait score with categories “not lame,” “moderately lame,” and “severely lame” (22). As we needed indicators that can be expressed as a percentage, only severe lameness was used in the ensuing steps. Integument alterations consist of both hairless patches and lesions/swellings. As both may have different causes, we chose to separate the two in the ensuing steps of this study.

### Step 2: Severity Scores

Median expert severity scores were highest for severe lameness (92, interquartile range = 90–97) and mortality (90, 69–100) followed by leanness (61, 50–71) and SCC > 400,000 (73, 43–80), and lowest for hairless patches (18–34) and wounds/swellings (40–58).

### Step 3: Calculating the WI and Analyzing Coherence With Expert Scores

**Figure 4:**
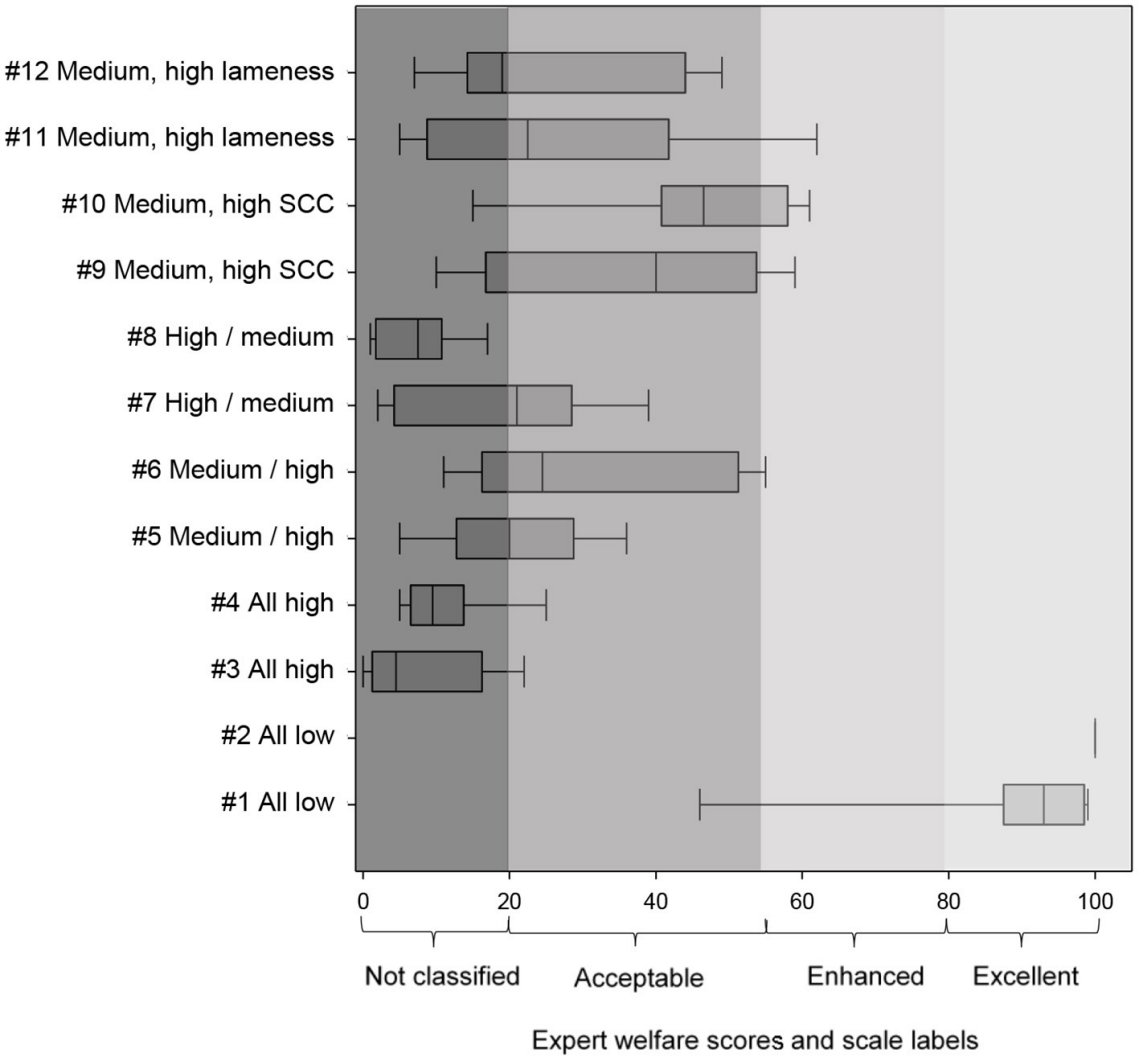
Medians and interquartile range (box) of the welfare scores allocated by experts (n = 14) to the 12 focus farms (confer Table 2) using a 0–100 tagged Visual Analog Scale. Whiskers: data within 1.5 × the interquartile range. Higher scores imply better welfare. QBA, Qualitative Behavior Assessments. Highest scores belonged to the top 5% of herds in the European dataset (n = 491), medium between 40 and 60%, and lowest scores were from the lowest 5% of herds. ^1^“% of too lean cows,” “SCC > 400,000,” and “number of hairless patches” were high, ^2^“% of cows with lesions,” “% of cows with severe lameness,” and “% of mortality” were high.

Welfare scores as indicated by the experts followed the patterns anticipated for the 12 focus herds (Figure 4). Herds 1 and 2, with a low prevalence for all measures, received a good score, while herds 3 and 4, with high prevalence for all measures (indicating poor welfare), received a bad score. Additionally, a high prevalence of the measure “severe lameness” while all other prevalences were medium (herds 11 and 12), lead to a lower expert score than when only “% of cows with SCC > 400,000” was high (herds 9 and 10), in line with the higher severity scores for lameness than SCC.

**Figure 5:**
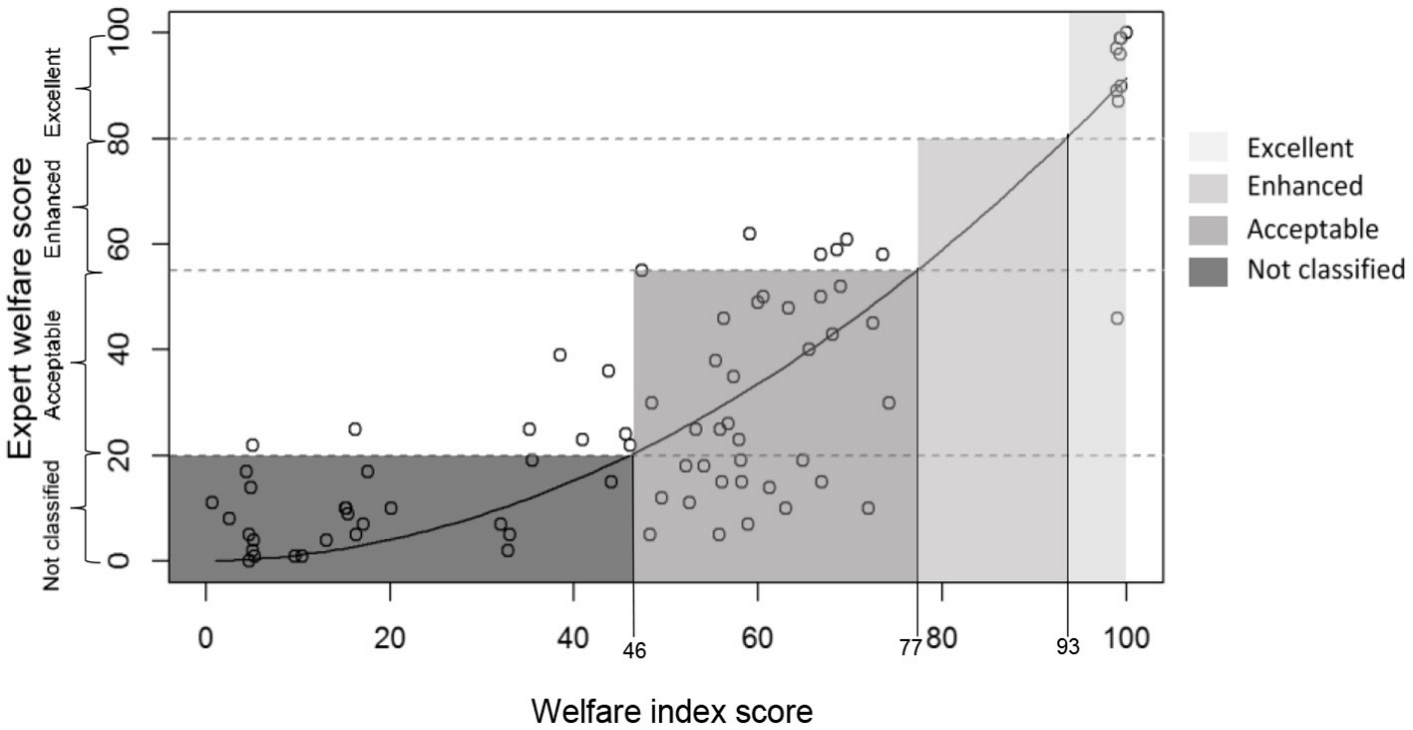
Expert (n = 14) welfare scores of the fictitious herds (n = 12) plotted against the calculated WI scores of these herds using no CRM, with best fitting quadratic curve (R^2^ = 0.91). Higher scores indicate better welfare; category thresholds determined using the expert scores are indicated underneath the x-axis.

Quadratic models consistently achieved a higher R^2^ than linear models. Using R^2^ as a primary metric for agreement, the quadratic model with no CRM (i.e., full compensation) provided the best fit with the experts' scores (R^2^ = 0.91, F = 401.4, Figure 5). For the 20 models with highest R^2^, the full compensation model was ranked first for four other agreement metrics (AIC = 688.4, mean absolute difference = 18.23, RMSE = 24.46, LOA = 46.21) and third for ICCC (0.737). The mean rank for all six metrics was also lowest (i.e., best) for the full compensation model (rank = 1.29), followed by two discrete compensation reduction models (ranks = 2.57 and 3.64). We thus conclude that full compensation provides the best fit with expert opinion. As there is no evidence that a method of compensation reduction improves the fit with the expert scores, we can simplify the WI by removing Cm from the formula.

### Step 4: Interpreting the WI

Based upon expert scores in the different welfare categories, thresholds for the category “Not classified” ranged from 0 to 46, for “Acceptable” from 46 to 77, for “Enhanced” from 77 to 93, and for “Excellent” from 93 to 100 (Figure 5).

### Step 5: Exhaustiveness Check

The welfare measures that were selected are all mentioned in Nielsen et al. (30) as being associated with what they defined as being the “worst adverse effects” based on expert opinion and literature (Table 2). Some “adverse effects” and a single “worst adverse effect” (thermal discomfort) were not associated with any of our selection of measures.

## Discussion

Aiming for a better uptake by the milk industry, we followed five steps to develop a thoroughly revised and simplified version of the WQ protocol for monitoring the welfare of dairy cattle herds. The main focus was to improve the cost effectiveness of the protocol by collecting information on a limited number of key welfare indicators in a much shorter time. The time needed for a certified assessor to complete the protocol was reduced by a factor of 2–3. For example, using the estimated time needed to assess the various welfare measures listed in Welfare Quality (22, Table 12), an assessment of a herd of 100 cows takes approximately 6 h and 41 min with the original WQ protocol vs. 2 h and 42 min with our simplified protocol. Our simplified tool for monitoring and integrated labeling of dairy cattle welfare distinguishes itself from the original WQ protocol (and most other protocols) in four other important ways. First, the exclusive use of animal-based measures implies direct assessment of dairy cattle welfare (in contrast to the use of resource-based measures). Second, the simple and transparent integration formula for calculating overall welfare (WI) reduces the likelihood of unwanted side effects that are more likely to occur when using more complex aggregation procedures. The original WQ protocol is an example of a complex integration method that was innovative in its use of methods, where welfare measures are first integrated into 12 criteria scores and subsequently into 4 principle scores, which are then used to determine the overall welfare category (22). The welfare principles were separated to reflect different dimensions of welfare, and the complex integration methods were necessary to cope with the large number of measures included. However, an unintended consequence of the large number of measures and the method used to integrate them is that sensitivity of the overall welfare category to changes in individual welfare measures partly depends on the number of measures integrated into the criterion and principle scores (18, 21). For our revised protocol, we opted for a much simpler, but intuitively sensible and transparent, method of integration using a single formula in which the relative weights of the various measures directly reflect how severely they affect cattle welfare (as judged by the experts). Third, the WI was based upon, tested, and found to show high correspondence with expert opinion. Finally, the integrated WI being expressed on a continuous scale ensures a high degree of differentiation, which enables detection of relatively small differences between (or within) herds. This implies that even small improvements in individual measures will lead to (slightly) higher integrated scores. Such a high degree of sensitivity is likely more motivating for farmers to implement on-farm welfare improvements than a (categorical) WI, which changes only in response to very drastic improvements.

The formula we eventually used for calculating WI also directly reflects the experts' opinion of how severely cattle welfare is affected by the various welfare issues that are quantified by the selected animal-based measures because the compensation-reduction term could be removed. Models for none of the compensation-reduction methods produced a better fit with the overall welfare scores given to the focus herds by the experts when compared to applying no compensation reduction. This implies that our expert consultation provided no justification to insert additional terms for calculating the WI so that the lowest measure scores are given additional weight relative to the other measure scores. Hence, we recommend using the simplest formula for WI (i.e., without the Cm term, which is assumed to equal one). The simplification of the original WQ protocol into our WI has recently been shown to result in a better match with five other (i.e., non-WQ based) dairy herd welfare assessment metrics used in the Netherlands and with the consensus herd welfare score given by at least five dairy cattle veterinarians that visited the farms on a regular basis (40). These findings thus provide some support for an improved concurrent and consensus validity, respectively, of the WI as compared to the original WQ overall categorization.

The time reduction and simplification of the protocol inevitably comes at the expense of the comprehensiveness of the assessment. It should be borne in mind, therefore, that the aim of the revised protocol is not to detect *all* possible adverse or favorable effects on dairy cattle welfare, which we consider virtually impossible. Instead, we focused on an index that reflects the worst adverse effects on welfare (according to literature and experts). Incorporating an extensive list of welfare measures would complicate step 3 of the process (comparing expert scores with WIs) possibly leading to information overload. This occurs when people are unable to distinguish relevant from irrelevant information when presented with too much information (41, 42). As we are aware that our limited selection of measures is not sensitive to all possible adverse welfare effects, we strongly advice to use a disclaimer indicating which adverse effects may not be detected by the current selection of measures. This approach may be considered as more fair than claiming exhaustiveness, which, in our opinion, is close to impossible anyway. The proposed WI does enable detection of all the *worst* adverse effects on dairy cattle welfare according to Nielsen et al. (30). Nielsen et al. (30) selected the worst adverse effects from a list of adverse effects on dairy cattle welfare, based upon EFSA reports. Although this list was not assessed for comprehensiveness—this remains to be validated by future research—our current selection of welfare measures likely lacks sensitivity for documenting some additional (not-worst) adverse effects (i.e., reproductive disorders; thermal discomfort; pain, fear, and frustration; abomasal displacement; respiratory distress/pain; other adverse effects related to diseases and other adverse effects related to injuries). We note that the measures that were retained in the simplified protocol focus on the impairment of the health and physical condition of the animals. This focus partly reflects the approach in the original WQ protocol, which includes only a single animal-based measure that could (arguably) provide information on positive affective state, namely, the Qualitative Behavior Assessment (QBA). The experts, however, allocated the least importance to this measure, which probably reflects reservations about the reliability or validity of this measure. In our opinion, this reflects a more general problem in animal welfare monitoring that there is a need to develop feasible, reliable, and valid measures that better document the behavioral needs and (negative as well as positive) affective states of the animals. Indeed, Knierim et al. (43) also questioned whether health-centered welfare assessment protocols that are implemented in the dairy industry, such as the US-based FARM program (https://nationaldairyfarm.com/farm-animal-care-version-4-0/) or the UK-based AssureWel protocol (http://www.assurewel.org/dairycows) sufficiently take societal expectations into account, which often relate to naturalness for dairy cattle. Perhaps with the rapid advancements in the use of automated sensor technologies for monitoring livestock behavior and condition, such information may be incorporated in welfare assessment protocols in the future (44–46). Such measures could be added to the protocol by using the step-wise approach we proposed. Such additions would make the assessment more comprehensive and hedonic (47, 48) but at the expense of simplicity and logistic feasibility.

Experts were stimulated in the survey to take validity and reliability of the WQ measures into account for ranking of the welfare measures. However, it still may be questioned whether validity and reliability of all selected measures are truly adequate. For example, mortality rate is based on herd records of which reliability has barely been documented. As is the case for any welfare assessment protocol, it is important to strive for high reliability of the measures by training observers to achieve high test–retest, inter- and intraobserver reliability, and by unbiased sampling of animals.

Categorical differentiation between herds (i.e., welfare categorization) is useful to interpret the WI in terms of which scores indicate farms of poor or excellent welfare. In addition, such welfare categories may be used for labeling purposes to identify farms of varying welfare levels. In the current study, we determined thresholds based upon expert scores in the different welfare categories for the 12 focus herds. These thresholds are only indicative, given the limited number of herds and experts that they are based on.

Two main inputs were used in the current study: expert opinion and a European database of selected welfare measures' prevalence. As expert opinion was vital in the current study, we used stringent criteria to select experts. While this limited the number of experts who could participate, it also ensured adequate knowledge about dairy cattle welfare and the welfare measures concerned. Still, it would be relevant to test whether outcomes (in terms of selected welfare measures, severity scores, and correspondence with expert opinion) would be similar with a different composition or type of experts. Similarly, it would be interesting as well to test whether another setting [e.g., a workshop to achieve consensus like in Rodenburg et al. (27)] would affect the outcomes. Moreover, it could be argued that in order for the protocol to be perceived as being of high quality and hence be advocated by the industry, other stakeholders ought to have been involved in the selection of measures and the way these are integrated. We opted to base our current study on the opinion of scientific experts who are knowledgeable about the WQ measures for assessing cattle welfare, rather than on other stakeholders who do not have the same level of expertise (e.g., consumers) or who might have non-scientific motivations to bias the aggregation outcome in one way or another (e.g., milk industry). In our opinion, such in-depth knowledge was essential important for making well-informed decisions about which measures to retain, allocating the severity scores and allocating overall welfare scores to the focus herds. It could be verified in a follow-up study whether consumers and other stakeholders accept or refute the authority and outcome of these scientific expert judgements. If this would reveal important discrepancies, we would face the dilemma of increasing social acceptance either by adapting the protocol to better accord with stakeholder opinion or by better clarifying and explaining the decisions, outcomes, and credibility of the scientific experts to the stakeholders.

The second important, and rather unique, input used in the current study was the database containing prevalence data on the selected measures of 491 European dairy herds. This dataset allowed selected experts to benchmark results of the focus farms based on a wide range of data, which supported them in allocating welfare scores that can realistically be attained on commercial farms in Europe. Such a large database on other (non-WQ) measures where a uniform protocol was used may be hard to attain. As both the experts and the dataset were European, caution is required when applying the protocol to dairy herds in other parts of the world (where the welfare challenges for cattle may be different).

## Conclusions

The stepwise approach employed in the current study led to thorough revision of the WQ protocol for on-farm monitoring of dairy herd welfare that is more user-friendly, more time efficient, and exclusively relies on key animal-based welfare measures (lameness, leanness, mortality, hairless patches, lesions/swellings, and somatic cell count) that are integrated into a highly differentiating, transparent, and continuous welfare index. In addition, the resulting WI is highly coherent with expert opinion. Although the reduction in the number of welfare measures reduces the comprehensiveness of the assessment, the current selection of six welfare measures are associated with all the worst adverse effects for dairy cattle welfare as identified by Nielsen et al. (30). Nevertheless, the integrated welfare index should be accompanied with a disclaimer that lists adverse and favorable effects that cannot be detected adequately by the current selection of measures. However, the proposed method is flexible such that measures can be replaced or added as deemed desirable by repeating the proposed steps.

## Data Availability Statement

The raw data supporting the conclusions of this article will be made available by the authors, without undue reservation.

## Ethics Statement

Written informed consent for participation was not required for this study in accordance with the national legislation and the institutional requirements. Data recorded in the study is reported anonymously.

## Author Contributions

FT and SG wrote the paper. BA did the statistical analyses. WV, LL, JB, FT, and SG conceptualized the study. AB, FE, MH, MKi, LM, and MKj provided data. All authors have proof-read earlier manuscripts.

## Conflict of Interest

The authors declare that the research was conducted in the absence of any commercial or financial relationships that could be construed as a potential conflict of interest.
